# Molecular Dynamics Free Energy Simulations Reveal the Mechanism for the Antiviral Resistance of the M66I HIV-1 Capsid Mutation

**DOI:** 10.3390/v13050920

**Published:** 2021-05-15

**Authors:** Qinfang Sun, Ronald M. Levy, Karen A. Kirby, Zhengqiang Wang, Stefan G. Sarafianos, Nanjie Deng

**Affiliations:** 1 Center for Biophysics and Computational Biology and Department of Chemistry, Temple University, Philadelphia, PA 19122, USA; tum97117@temple.edu; 2 Laboratory of Biochemical Pharmacology, Department of Pediatrics, Emory University School of Medicine, Atlanta, GA 30322, USA; karen.kirby@emory.edu (K.A.K.); stefanos.sarafianos@emory.edu (S.G.S.); 3 Children’s Healthcare of Atlanta, Atlanta, GA 30322, USA; 4 Center for Drug Design, College of Pharmacy, University of Minnesota, Minneapolis, MN 55455, USA; wangx472@umn.edu; 5 Department of Chemistry and Physical Sciences, Pace University, New York, NY 10038, USA

**Keywords:** HIV-1 capsid, drug resistance mutation, free energy simulation, molecular dynamics, protein reorganization

## Abstract

While drug resistance mutations can often be attributed to the loss of direct or solvent-mediated protein−ligand interactions in the drug-mutant complex, in this study we show that a resistance mutation for the picomolar HIV-1 capsid (CA)-targeting antiviral (GS-6207) is mainly due to the free energy cost of the drug-induced protein side chain reorganization in the mutant protein. Among several mutations, M66I causes the most suppression of the GS-6207 antiviral activity (up to ~84,000-fold), and only 83- and 68-fold reductions for PF74 and ZW-1261, respectively. To understand the molecular basis of this drug resistance, we conducted molecular dynamics free energy simulations to study the structures, energetics, and conformational free energy landscapes involved in the inhibitors binding at the interface of two CA monomers. To minimize the protein−ligand steric clash, the I66 side chain in the M66I−GS-6207 complex switches to a higher free energy conformation from the one adopted in the apo M66I. In contrast, the binding of GS-6207 to the wild-type CA does not lead to any significant M66 conformational change. Based on an analysis that decomposes the absolute binding free energy into contributions from two receptor conformational states, it appears that it is the free energy cost of side chain reorganization rather than the reduced protein−ligand interaction that is largely responsible for the drug resistance against GS-6207.

## 1. Introduction

**Figure 1:**
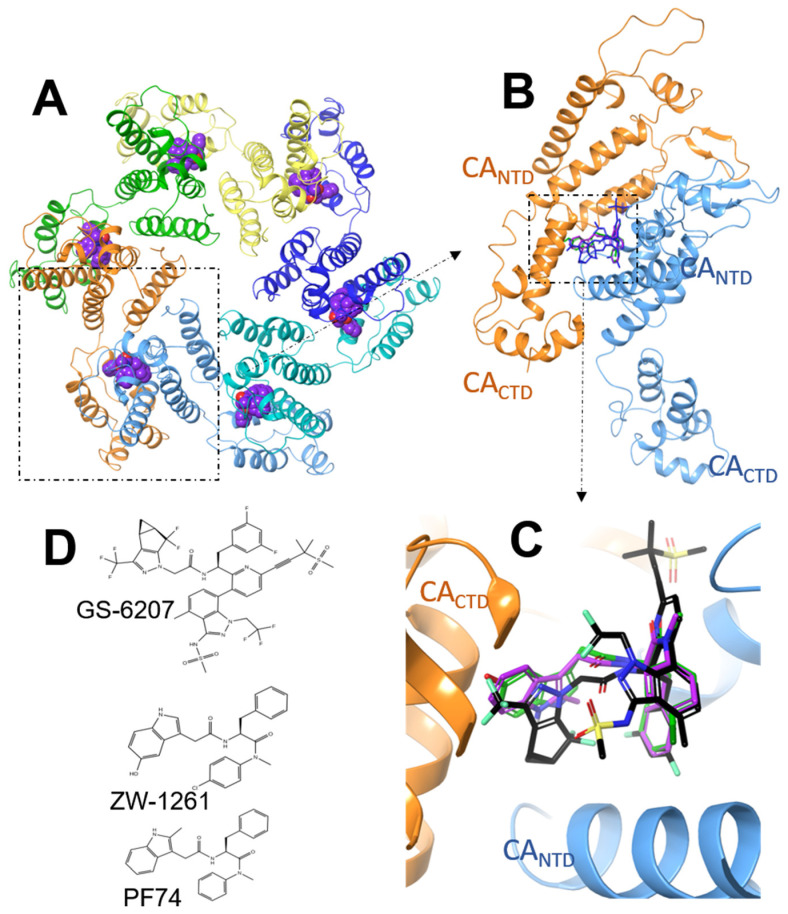
(**A**): Crystal structure of PF74 (purple) bound at the dimeric interface of a CA hexamer. (**B**): Crystal structure of GS-6207 (black), ZW-1261 (green), and PF74 (purple) bound at the dimeric interface of two CA monomers. (**C**): The binding site of the ligands at the NTD−CTD interface in a CA dimer. (**D**): Chemical structures of GS-6207, ZW-1261, and PF74.

In recent years, HIV-1 CA has emerged as a major drug target for antiviral therapeutic development [1,2,3]. CA not only acts as the building block of the HIV-1 capsid core formation but also interacts with several host factors [4,5,6,7,8]. Small molecule agents with different chemotypes have been developed in recent years to disrupt the CA biological functions in the virus replication cycle [9,10,11,12]. Among them, GS-6207 exhibits antiviral activity at picomolar concentrations and is metabolically stable, which makes it highly promising as a potential long-acting agent for use in antiviral therapy [3,11,12,13]. However, recent studies have identified several amino acid mutations in the CA binding site that confer drug resistance to GS-6207 [13,14]. Among them, M66I causes the greatest reduction in GS-6207 antiviral activity by up to ~84,000-fold compared to WT HIV-1 CA [14]. In contrast, the same mutation causes a resistance of only 83-fold to PF74 and 68-fold to the PF74 derivative ZW-1261 [5,9,15] (Figure 1 and Table 1). As a result, although the EC_50_ of ZW-1261 against WT HIV-1 is higher than that of GS-6207, the EC_50_ values of these compounds against HIV-1 M66I are comparable (Table 1). The molecular mechanism of resistance imparted by M66I and its differential effects on the binding of these antivirals is not immediately obvious from the crystal structures of the CA−compound complexes.

All-atom molecular dynamics simulations (MD) can provide detailed information on the energetics, solvation, and conformational dynamics of molecular recognition [16]. We have previously used this approach to study HIV-1 integrase mechanisms of drug resistance [17,18,19,20,21]. To understand the detailed mechanisms of HIV-1 drug resistance through the M66I CA mutation at the atomic level, we applied MD free energy simulations in explicit solvent to study the energetics, thermodynamics, and conformational dynamics of the binding of different antivirals at the shared compound-binding site. The calculated relative binding free energies correlated well with the EC_50_ measurements. The results showed that to minimize steric clashes due to the binding of GS-6207, the I66 side chain of the HIV-1 CA mutant undergoes conformational changes away from the apo protein and adopts a rotamer state that is about 3.6 kcal/mol higher in free energy. In contrast, the binding of GS-6207 with the wild-type CA does not lead to significant protein conformational changes. Using an analysis that decomposes the absolute binding free energy into contributions from two receptor conformational macrostates, we found that the free energy of the protein side chain reorganization plays a dominant role in determining the mechanism of M66I resistance to GS-6207. This study provides mechanistic insight into the differential effects of the M66I resistance mutation against the HIV-1 capsid-targeting compounds and could help to inform the design of a new generation of antivirals with improved resistance profiles.

## 2. Methods and Materials

### 2.1. Experimental Antiviral Activity Assay (EC_50_)

The wild-type (WT) laboratory HIV-1 strain, HIV-1_NL4-3_ [22], was produced using a pNL4-3 vector that was obtained through the NIH AIDS Reagent Program, Division of AIDS, NIAID, NIH. The mutation M66I in the capsid region was introduced into this vector by site-directed mutagenesis. WT and M66I HIV-1_NL4-3_ viruses were generated by transfecting HEK 293FT cells in a T75 flask with 10 µg of the pNL4-3 vector and FuGENE^®^HD Transfection Reagent (Promega, Madison, WI, USA). Supernatant was harvested 48–72 h post-transfection and transferred to MT2 cells for viral propagation. The virus was harvested when syncytia formation was observed, after approximately 3–5 days. The viral supernatant was concentrated using 8% *w*/*v* PEG 8000 overnight at 4 °C, followed by centrifugation for 40 min at 3500 rpm. The resulting viral-containing pellet was concentrated 10-fold by resuspension in DMEM without FBS and stored at −80 °C. The anti-HIV-1 activity of PF74 and ZW-1261 against the WT and M66I HIV-1 viruses was examined in TZM-GFP cells [23,24]. The potency of viral inhibition was based on the compounds’ inhibitory effect on the viral LTR-activated GFP expression compared with that of compound-free (DMSO) controls. TZM-GFP cells were plated at a density of 1 × 10^4^ cells per well in a 96-well plate. Twenty-four hours later, the medium was replaced with increasing concentrations of the compound. Twenty-four hours post treatment, cells were exposed to WT or M66I HIV-1 (Multiplicity Of Infection (MOI) = 1). After incubation for 48 h, the anti-HIV-1 activity was assessed by counting the number of GFP positive cells on a Cytation ^TM^ 5 Imaging Reader (BioTek, Winooski, VT, USA), and 50% effective concentration (EC_50_) values were determined. Antiviral assays were conducted in three technical replicates and repeated in at least three independent experiments.

### 2.2. Crystallization, Data Collection and Structure Determination

Native, full-length wild-type HIV-1 CA in a pET11a vector was expressed and purified, as previously described [1,25]. Hexagonal CA crystals grew in specific conditions, as previously described [1]. CA crystals were soaked in a solution containing ZW-1261 (1.25 mM final concentration with 5% DMSO) for ~4 h, then briefly transferred to a solution containing 22% glycerol for cryoprotection before flash freezing in liquid nitrogen.

Data were collected on a Dectris Eiger 16 M detector at Advanced Photon Source (APS) beamline 22-ID at the Argonne National Laboratory. Data were processed to 2.7 Å using XDS [26,27], and indexed in the hexagonal space group P6 with unit cell dimensions *a*, *b* = 90.9 Å and *c* = 56.1 Å, and one CA molecule in the asymmetric unit. Data were analyzed using XTRIAGE, which determined that no twinning was present [28]. Initial phases were solved via molecular replacement with Phaser [29,30], using coordinates of a native full-length CA in complex with PF74 (PDB ID: 4XFZ) [1] as a starting model, with all ligands and solvent removed. The resulting model was refined using REFMAC5 [31]. The coordinates and ligand topology of ZW-1261 were generated using PRODRG [32]. ZW-1261 was built into the model using a difference Fourier map calculated in the absence of ligand. The CA/ZW-1261 model was improved through several iterative rounds of model building and refinement using Coot [33,34] and REFMAC5 [31], respectively. The final model was validated using MOLPROBITY [35,36]. The final structure factors and coordinates have been deposited into the Protein Data Bank and are available under accession code 7M9F. Data collection, processing, and refinement statistics are provided in Supplementary Table S1. Further examination of this structure will be presented in another manuscript.

### 2.3. System Setup and Simulation Details

The starting structure of GS-6207 in complex with a CA dimer was extracted from the crystal structure of the complex of GS-6207 with a cross-linked CA hexamer (PDB ID: 6V2F) [13]. The starting complex structures of PF74 and ZW-1261 with a CA dimer were obtained from the corresponding crystal structures of the two ligands with native CA hexamers (PDB ID: 4XFZ and PDB ID: 7M9F, respectively) [1]. The structure of the M66I-GS-6207 complex used in the MD simulation in Figure 5 was obtained from FEP simulations using the FEP+ program [37] from the Schrödinger Suite that mutated MET66 into ILE. Starting from the crystal structure of the wild-type CA in complex with the GS-6207, a series of FEP simulations were performed to alchemically “mutate” the MET66 side chain into ILE. The structure of the M66I-GS-6207 complex corresponded to the final state (λ=1) of the FEP simulation.

The FEP+ program [37] from the Schrödinger Suite 2020-4 [38] was also used to calculate the relative binding free energies of the wild-type and mutant M66I. The wild-type complexes were prepared with Protein Preparation Wizard [39]. For every wild-type complex and its corresponding mutant, the FEP Protein Mutation for ligand Selectivity GUI [37,38] in Maestro Suite [40] was applied to build a perturbation map. The initial structure of the apo mutant was prepared by deleting the ligand from the corresponding complexes and equilibrating the resulting structure in a solution for 20 ns, using the Desmond module.

The OPLS3e force field was used to model proteins and ligands. [41] Torsion parameters were checked for all ligand fragments using a Force Field Builder. A 10 Å cubic box filled with ~31,300 SPC water [42] was used for the complex and solvent perturbation leg (wild-type and mutant apo protein). Additional Na+ and Cl− ions were added to obtain 0.15 M NaCl in the simulation box. The number of alchemical λ windows was set to 12 by default to connect the wild-type and the mutant states. For each λ window, the production MD was run for 15 ns in the NPT ensemble. The Bennett acceptance ratio method [43] (BAR) was used to calculate the free energy differences. The mutational free energy calculations were performed in triplicate by initializing the MD with different random seeds. The reported relative binding free energies were calculated as the average of the three ΔΔG from independent simulations.

The metadynamics [44] module implemented in the Desmond software package was used to explore the complex free-energy surface of the $\chi_{1}$ dihedral of the I66 in the apo structure of the M66I mutant. Three independent, 50 ns well-tempered metadynamics [45] simulations were run with the initial Gaussian hill height and well-tempered parameters set at 0.03 and 2.4 kcal/mol, respectively. The metadynamics data were processed utilizing the Metadynamics Analysis module (Schrödinger, LLC, New York, NY, USA) to compute the free energy surface.

## 3. Results and Discussion

In this section, we show that the loss of binding affinity for the GS-6207 due to the M66I CA mutation can be largely explained by the fact that the GS-6207 forces the M66I CA mutant to undergo a conformational switch to a higher free energy state, while such a conformational change is absent in the case of GS-6207 binding to the wild-type CA or when the mutant CA binds with PF74 and its derivative ZW-1261. By considering the total binding free energy as contributions from different receptor macrostates (see Equation (1) below), we demonstrate the important role played by the free energy of the protein reorganization in ligand binding.

### 3.1. The Calculated Relative Binding Free Energies Capture the Trend of the Biological Assays

**Figure 2:**
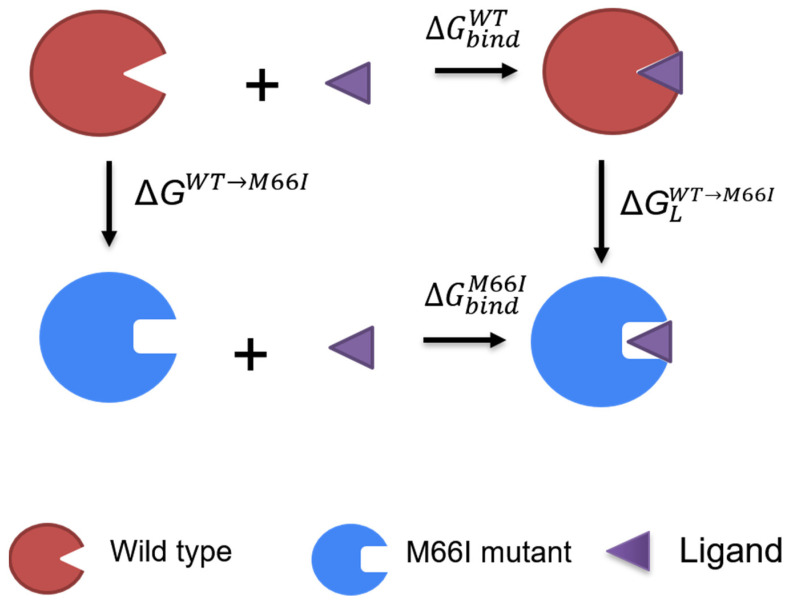
The thermodynamic cycle used for computing the changes in the binding free energy caused by the M66I mutation in the binding of the ligand to the CA dimer.

Table 1 shows the experimental EC_50_ results for PF74, ZW-1261, and GS-6207 against the wild-type and the M66I mutant HIV-1 viruses. While the M66I mutation reduces the potency of GS-6207 by up to ~84,000-fold, the activities of ZW-1261 and PF74 are only reduced by 68-fold and 83-fold, respectively. To test whether the all-atom MD free energy model can capture this trend, we examined the changes in the binding free energy caused by the M66I mutation during the binding of the three antivirals to the CA dimer by using FEP (free energy perturbation) to calculate the relative binding free energy $\Delta \Delta G_{bind}^{WT\to M66I}$ for each of the antivirals. In the thermodynamic cycle shown in Figure 2, $\Delta \Delta G_{bind}^{WT\to M66I\;}\equiv \Delta G_{bind}^{M66I}-\Delta G_{bind}^{WT}={\Delta G}_{L}^{WT\to M66I}-{\Delta G}^{WT\to M66I}$, i.e., the relative binding free energy $\Delta \Delta G_{bind}^{WT\to M66I}$ can be obtained by the two vertical legs, instead of by simulating the physical binding process (the two horizontal legs in Figure 2), which is much more difficult to converge. As seen in Table 2, the results of the FEP calculations estimate that the binding affinities of GS-6207, ZW-1261, and PF74 are reduced by 367-fold, 43-fold, and 58-fold, respectively, which correlates well with the experimentally determined EC_50_ values. Note that complete numerical agreement is not expected here, since the calculation measures K_D_ rather than EC_50_, even though the two generally correlate with each other. The results show that the all-atom free energy model with an explicit solvent successfully accounts for the effect of the M66I mutation on the change in the binding of the three antivirals to the CA dimer. Below, we report the results of the further analysis of the MD free energy simulations to gain insight into the mechanism of the M66I resistance mutation.

### 3.2. The Binding of GS-6207 to the M66I CA Mutant Forces the I66 Side Chain to Adopt a Higher Free Energy Rotamer State to Avoid Steric Clashes

**Figure 3:**
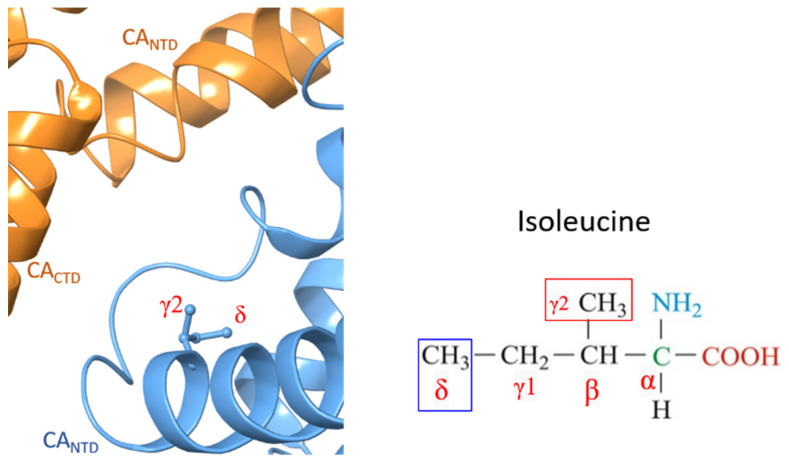
Structure of the apo pocket of the M66I CA mutant from MD. Isoleucine 66 is illustrated by the ball and stick. One CA monomer is illustrated by the blue cartoon, while the neighboring CA monomer is illustrated by the orange cartoon.

**Figure 4:**
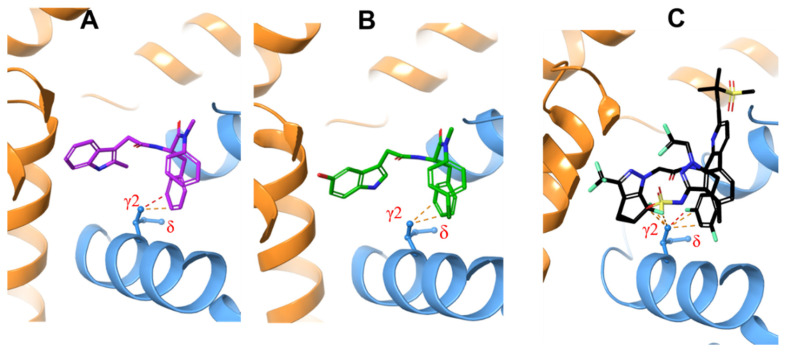
PF74 (**A**) in purple, ZW-1261 (**B**) in green, and GS-6207 (**C**) in black are inserted into the MD structure of the apo M66I CA mutant pocket by superimposing the protein in the holo M66I with that of the apo M66I. The atomic clashes are illustrated by yellow dashed lines.

To understand why the M66I mutation causes such a significant loss of binding affinity to GS-6207 and why the loss of affinity is bigger than it is for ZW-1261 and PF74, we first examine the apo structure of the M66I mutant (Figure 3), which is obtained by manually removing the ligand from the M66I−GS-6207 complex and running an MD simulation on the apo protein in a solution for 20 ns. The structure of the M66I−GS-6207 complex corresponds to a representative structure in the final state following the FEP simulation that mutates the MET66 in the WT−GS-6207 complex to ILE using the FEP+ module of Schrodinger Inc.; see details in the Method and Materials Section. We find that in the apo mutant structure, the I66 side chain adopts a conformer in which the γ2 carbon is above the δ carbon in the orientation adopted in Figure 3. This corresponds to the lowest free energy rotamer of the I66 side chain (see also Figure 9B below). We then manually place the three antiviral ligands into this apo pocket by superimposing the protein in the corresponding holo mutant structures onto that in the apo mutant structure. The resulting complex containing GS-6207 shows significantly more extensive atomic clashes with the γ2 carbon of I66 compared with structures containing ZW-1261 and PF74 (see Figure 4).

**Figure 5:**
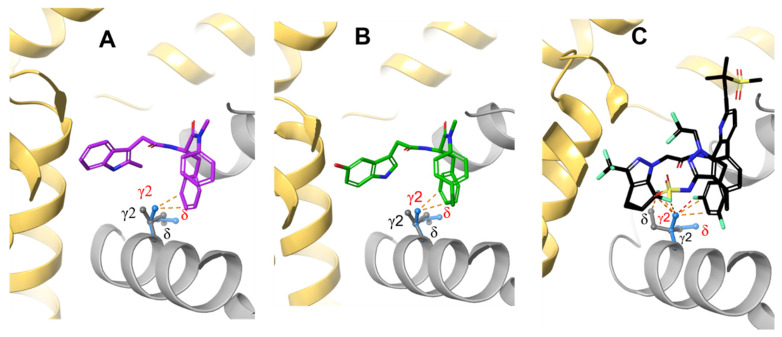
Representative MD snapshots of (**A**) PF74 (purple); (**B**) ZW-1261 (green); and (**C**) GS-6207 (black) in complex with the M66I CA mutant (gray and yellow cartoon, the I66 side chain is depicted by gray sticks). For comparison, the I66 side chain of the apo M66I mutant is depicted by light blue sticks in all panels.

**Figure 6:**
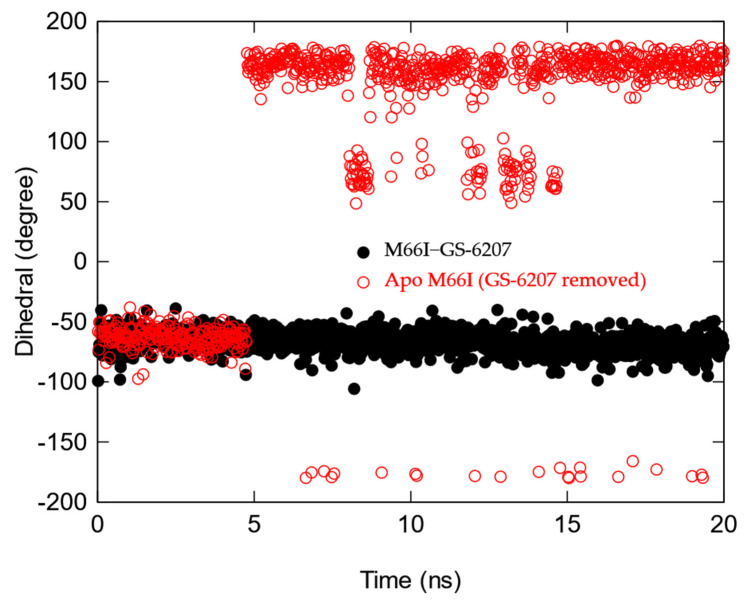
Time course of the $\chi_{1}$ torsion in the side chain of I66 of the M66I CA mutant after the GS-6207 is manually removed (red), M66I−GS-6207 complex (black).

**Figure 7:**
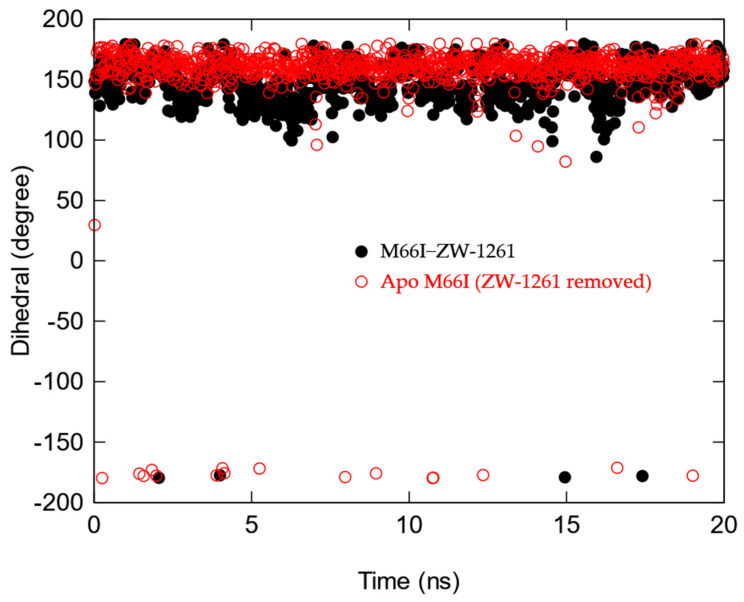
Time course of the $\chi_{1}$ torsion in the side chain of I66 of the M66I CA mutant after the ZW-1261 is manually removed (red), M66I−ZW-1261complex (black).

**Figure 8:**
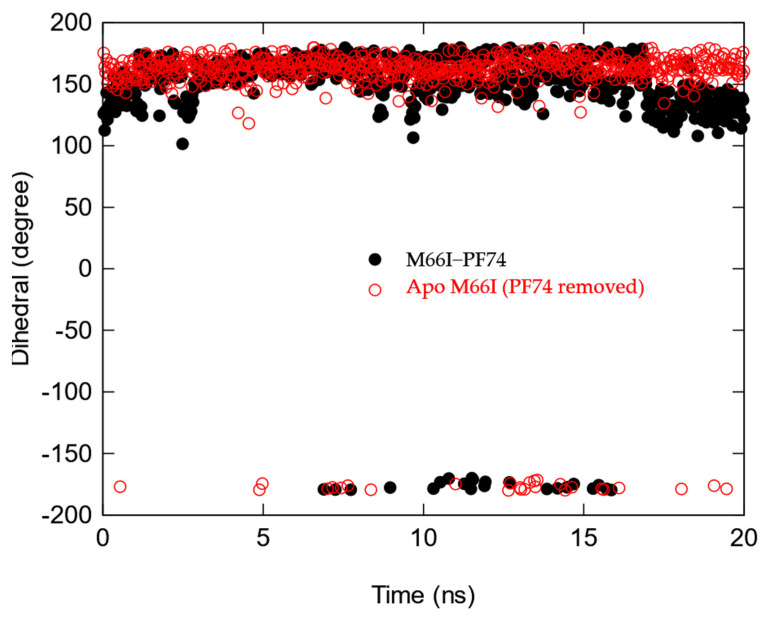
Time course of the $\chi_{1}$ torsion in the side chain of I66 of the M66I CA mutant after the PF74 is manually removed (red), M66I−PF74 complex (black).

We then compared the MD structures of the M66I CA mutant in complex with GS-6207, ZW-1261, and PF74 (Figure 5). While the I66 side chain in the M66I−ZW-1261 or PF74 complexes essentially maintained the same rotamer state as that of the one in the apo protein in Figure 3, in the M66I−GS-6207 complex the I66 side chain moved to a new, higher-energy rotamer, with the γ2 carbon and δ carbon switching positions. To show that this new conformer in the M66I−GS-6207 complex was indeed of s higher free energy, we performed the following sets of MD simulations: (1) starting from the structure of the M66I−GS-6207 complex but with the ligand removed from the binding pocket; (2) starting from the structure of the M66I−GS-6207 complex; (3) starting from the structures of the M66I−ZW-1261 and M66I−PF74 complexes with the ligands removed from the binding pocket; and (4) starting from the structures of the M66I−ZW-1261 and M66I−PF74 complexes. Figure 6, Figure 7 and Figure 8 show the $\chi_{1}$ dihedral angle of I66 as a function of the simulation time during a 20 ns MD for each of the four simulations. As seen in Figure 6, when GS-6207 is removed from its complex with the M66I CA mutant, after ~5 ns the $\chi_{1}$ torsion of the I66 side chain spontaneously switches to the conformation in the apo mutant (shown in red) and maintains that rotamer state during the remaining ~15 ns of the MD trajectory. Although this χ1 torsion occasionally visits conformations in the 50~100 degree region during the ~15 ns MD, such metastable states are not seen in the longer MD simulation (data now shown). In contrast, in all other simulations the $\chi_{1}$ torsion of I66 remains in the same starting rotamer state during the entire MD.

**Figure 9:**
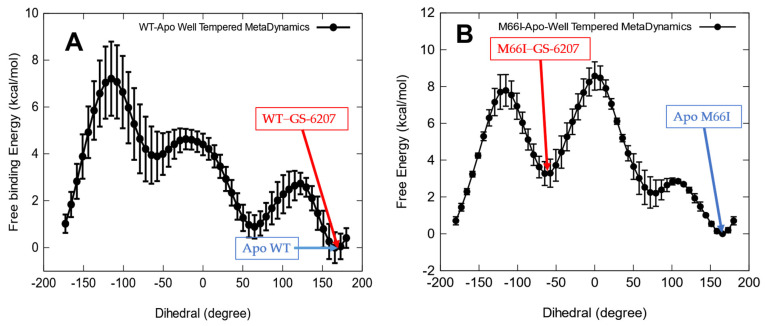
Free energy profiles along the $\chi_{1}$ dihedral angle of the side chain of residue 66 in (**A**) apo wild-type CA and (**B**) apo M66I mutant.

To quantitatively estimate the free energy change associated with the GS-6207-induced conformational reorganization, we calculated the free energy profiles using well-tempered metadynamics simulations along the $\chi_{1}$ of the side chain of residue 66 in the apo forms of the wild-type CA and the M66I mutant (Figure 9). As shown in Figure 9B, the global free energy minimum ($\chi_{1}\approx 160{}^{\circ}$) in the apo M66I CA coincides with the rotamer observed in the M66I−ZW-1261 and M66I−PF74 complexes (see Figure 7 and Figure 8). In contrast, the rotamer populated in the M66I−GS-6207 complex ($\chi_{1}\approx -70{}^{\circ}$, Figure 6) has a higher conformational free energy of $\Delta G_{I66}\left(160{}^{\circ}\to -70{}^{\circ}\right)\approx 3.6\;\pm 0.6\;\mathrm{kcal}/\mathrm{mol}$ than the global free energy minimum of $\chi_{1}\approx 160{}^{\circ}$ in the apo M66I CA structure (Figure 9B). Importantly, this conformational free energy difference $\Delta G_{I66}\left(160{}^{\circ}\to -70{}^{\circ}\right)$ correlates well with the relative binding free energy $\Delta \Delta G_{bind}^{WT\to M66I\;}=3.52\;\mathrm{kcal}/\mathrm{mol}$ caused by the M66I CA mutation for GS-6207 binding (Table 2).

While Figure 9B shows that the binding of GS-6207 to the M66I mutant induces a side chain conformational change to a higher free energy rotamer, this is not the case for the wild-type CA. As seen in Figure 9A, the same MET66 side chain rotamer state is populated in both the apo form and the WT−GS-6207 complex. In the next section, we show more quantitatively that this contrasting behavior of the presence and absence of a ligand-induced protein side chain reorganization in the mutant and wild-type CA is key to understanding the M66I resistance to GS-6207.

### 3.3. Decomposing the Absolute Binding Free Energy into Receptor Conformational States Suggests the Dominant Role of Protein Side Chain Reorganization in the Drug Resistance Mutation

Based on these observed conformational properties of the binding site, we explore the physical reason for the loss of the binding affinity of GS-6207 due to the M66I mutation. As shown in Figure 9B, the binding site in the M66I CA mutant exists in two conformational states, A (χ1=160°) and B (χ1=−70°), with their apo state occupancies $P_{A}\gg P_{B}$, since $\Delta G_{I66}\left(160{}^{\circ}\to -70{}^{\circ}\right)\approx 3.6\;\mathrm{kcal}/\mathrm{mol}$.

In general, the total absolute binding free energy can be written in terms of the contributions from each of the receptor conformational states [46,47]:

(1) $$\Delta G_{bind}=-kT\ln\left(P_{A}e^{-\Delta G_{bind}^{A}/kT}+P_{B}e^{-\Delta G_{bind}^{B}/kT}\right),$$

where $\Delta G_{bind}^{A}$ and $\Delta G_{bind}^{B}$ are the intrinsic binding free energies of the ligand for the conformational states A and B, respectively.

Here, since GS-6207 binding to state A of the M66I mutant results in a steric clash (Figure 4), $\Delta G_{bind}^{A}$ is highly unfavorable compared with $\Delta G_{bind}^{B}$. Therefore,

(2) $$\Delta G_{bind}\left(M66I-\mathrm{GS}-6207\right)\approx -kT\ln\left(P_{B}e^{-\Delta G_{bind}^{B}/kT}\right)=\Delta G_{bind}^{B}\left(M66I-\mathrm{GS}-6207\right)-kT\ln P_{B}.$$

$P_{A}\gg P_{B}$,$-kT\ln P_{B}$ approximately represents the protein side chain reorganization free energy $\Delta G_{I66}\left(160{}^{\circ}\to -70{}^{\circ}\right)$, and according to Figure 9B, $-kT\ln P_{B}\approx \Delta G_{I66}\left(160{}^{\circ}\to -70{}^{\circ}\right)=3.6\;\mathrm{kcal}/\mathrm{mol}$. When comparing this value with the relative binding free energy caused by the M66I CA mutation $\Delta \Delta G_{bind}^{WT\to M66I\;}=3.52\;\mathrm{kcal}/\mathrm{mol}$ (Table 2) and noting that the GS-6207 binding to the wild-type CA is not accompanied by a shift in the protein conformational states (Figure 9A), it is easy to show that the intrinsic binding free energy when the M66I mutant is restrained to the B state, $\Delta G_{bind}^{B}\left(M66I-\mathrm{GS}-6207\right)$, is close to the total absolute binding free energy for the wild-type CA, $\Delta G_{bind}\left(WT-GS-6207\right)$. Therefore, it is the protein side chain reorganization free energy $\Delta G_{I66}\left(160{}^{\circ}\to -70{}^{\circ}\right)$ that contributes the most to the relative binding free energy caused by the M66I CA mutation, $\Delta \Delta G_{bind}^{WT\to M66I}$.

## 4. Conclusions

While drug resistance mutations are often attributed to the loss of direct or solvent-mediated protein–ligand interactions in the drug-mutant complex [20], in this study we demonstrate that the molecular basis for the M66I resistance mutation of GS-6207 is mainly due to the free energy cost of the drug-induced protein side chain reorganization in the mutant protein. The experimental data show that the M66I mutation leads to an >84,000-fold reduction in the activity of GS-6207, a picomolar HIV-1 capsid-targeting antiviral, while the same mutation leads to only a 68-fold reduction in the activity of the newly designed antiviral ZW-1261 and a 83-fold loss in the activity of PF74. To understand the physical mechanisms of M66I resistance for these antivirals, we used all-atom molecular dynamics free energy calculations to study the energetics, structures, and conformational free energy profiles of the species involved in the binding of these antivirals to the wild-type CA and the M66I mutant. According to the free energy surfaces of the $\chi_{1}$ side chain dihedral angle, the I66 side chain in the M66I−GS-6207 complex populates a higher free energy rotamer state to minimize the steric clash. In contrast, the corresponding conformational changes are largely absent in the I66 of the M66I−ZW-1261 and M66I−PF74 complexes, and in the WT−GS-6207. By using a formula that decomposes the absolute binding free energy into contributions from two receptor conformational states, we show that the protein side chain reorganization plays a major role in the M66I resistance to GS-6207. This study provides plausible physical explanations for the mutation M66I that affects the three HIV-1 capsid-targeting antivirals differently at an atomic level, and could help to inform the design of new antivirals with improved resistance profiles.

## Figures and Tables

**Table 1:** EC_50_ measurements of PF74, ZW-1261, and GS-6207 against wild-type and M66I mutant viruses.

Ligand	Wild-Type (M)	M66I Mutant (M)	Fold Change WT → M66I
PF74	0.6 ^a^	>50	>83
ZW-1261	0.022	1.5	68
GS-6207 ^b^	3.1 × 10^−5^ − 4.5 × 10^−5^	0.1 − 3.8	3226 − 84,000

^a^ Ref. [9]. ^b^ Refs. [13,14].

**Table 2:** Changes in the ligand binding free energies caused by the M66I CA mutation for the antivirals PF74, ZW-1261, and GS-6207.

Ligand	$\Delta \Delta G_{bind}^{WT\to M66I}$ (kcal/mol)	Fold Change in K_D_
PF74	2.41 ± 0.40	58.2
ZW-1261	2.25 ± 0.49	43.6
GS-6207	3.52 ± 0.41	367.2

## Data Availability

All data are available from the corresponding author by e-mail request.

## Supplementary Materials

The following are available online at https://www.mdpi.com/article/10.3390/v13050920/s1, Table S1: X-ray data collection and refinement statistics for the native full-length HIV-1 CA/ZW-1261 complex (PDB ID: 7M9F).
