# Supplementary material for: Molecular Dynamics Free Energy Simulations Reveal the Mechanism for the Antiviral Resistance of the M66I HIV-1 Capsid Mutation
Source: Viruses. 2021 May 15;13(5):920. doi: 10.3390/v13050920 (PMC8156065; doi:10.3390/v13050920)
Supplement: Supplementary file 1 [file viruses-13-00920-s001.zip › viruses-1189168-supplementary.pdf]

Supplementary Table S1. X-ray data collection and refinement statistics for the native full-length HIV-1 CA / ZW-1261 complex (PDB ID: 7M9F).

**Data collection**	Beamline APS 22-ID
Wavelength (Å)	1.00000
Resolution (Å)	2.70 (2.70-2.77)[a]
Space group	P6
Cell dimensions	
a, c (Å)	90.9, 56.1
Observed reflections	52,178
Unique reflections	7,239
Redundancy	7.2 (6.1)
Completeness (%)	97.9 (91.1)
R_{meas}[b]	0.118 (0.84)
$CC_{1/2}$	99.6 (78.3)
Avg I/σ	13.6 (3.4)
**Refinement statistics**	
Resolution (Å)	45.71-2.70
No. of reflections (working)	6,881
No. of reflections (test)	356
R_{work}[c]	0.197
R_{free}[d]	0.253
Overall B value (Å^2)	70.0
Wilson B value (Å^2)	63.5
Ramachandran plot (%)[e]	
Favored	95%
Allowed	4.5
Disallowed	0.5
All-atom clashscore	4
RMSD Bond length (Å)	0.002
RMSD Angle (°)	0.994

[a] Values in parentheses are for the outer resolution shell.

[b] $R_{meas} = \Sigma_{hkl} \sqrt{\frac{n}{n-1}} \sum_{j=1}^{n} |I_{hkl,j} - < I_{hkl} > | \ / \ \Sigma_{hkl} \Sigma_j I_{hkl,j}$.

[c] $R_{cryst} = \Sigma_{hkl} |F_{obs} - F_{calc}| \ / \ \Sigma_{hkl} |F_{obs}|$.

[d] $R_{free} = R_{cryst}$, except 5% of the data excluded from the refinement.

[e] Evaluated by MolProbity (Chen *et al.*, Acta Cryst. D66, 2010, 12-21).
